# Supplementary material for: Patterns and Predictors of HIV Comorbidity among Adolescents and Young Adults in South Africa
Source: Int J Environ Res Public Health. 2024 Apr 9;21(4):457. doi: 10.3390/ijerph21040457 (PMC11050542; doi:10.3390/ijerph21040457)
Supplement: Supplementary file 1 [file ijerph-21-00457-s001.zip › ijerph-2880180-supplementary.pdf]

Table S1. Description of sample population by sex (unweighted)

	Total % (n) N=2723	Male % (n) n=1307	Female % (n) n=1416	*p*-Value
Age* (Median years and IQR)	19 (17–22)	19 (17–22)	19 (17–22)	0.22
Urban location	49.9 (1 360)	47.1 (616)	52.5 (744)	0.005
Province:				0.110
– Western Cape	5.5 (149)	4.8 (63)	6.1 (86)	
– Eastern Cape	13.6 (371)	14.9 (195)	12.4 (176)	
– Northern Cape	7.3 (200)	6.6 (86)	8.1 (114)	
– Free State	9.9 (270)	10.7 (140)	9.2 (130)	
– Kwa-Zulu Natal	18 (489)	17.1 (223)	18.8 (266)	
– North West	8.9 (242)	9.8 (128)	8.1 (114)	
– Gauteng	8.5 (231)	8 (104)	9 (127)	
– Mpumalanga	12.4 (338)	12.2 (159)	12.6 (179)	
– Limpopo	15.9 (433)	16 (209)	15.8 (224)	
Education level				<0.001
– Primary or less	10.4 (283)	13.9 (181)	7.2 (102)	
– Secondary complete	84.1 (2289)	81.9 (1071)	86 (1218)	
– Tertiary	5.6 (151)	4.2 (55)	6.8 (96)	
Employed	13.2 (358)	17.6 (230)	9.0 (128)	
Wealth index				**0.185**
– Quintile 1 (Poorest)	23.3 (634)	23 (301)	23.5 (333)	
– Quintile 2 (Poorer)	23.5 (640)	24.9 (326)	22.2 (314)	
– Quintile 3 (Middle)	23.1 (630)	23.9 (312)	22.5 (318)	
– Quintile 4 (Richer)	19.8 (540)	18.8 (246)	20.8 (294)	
– Quintile 5 (Richest)	10.3 (279)	9.3 (122)	11.1 (157)	

*Age in years. ‘Categorical variables tested using Chi-squared, continuous various tested using Wilcoxon.

Table S2. Description of sample population by age group (unweighted)

	Total % (n) N = 2723	15–19 year olds (n = 1435)	20–24 year olds (n = 1288)	
Sex (Male)	48.0 (1 307)	49.1 (705)	46.7 (602)	0.213
Location (Urban)	49.9 (1 360)	46.6 (669)	53.7 (691)	**<0.0001**
Province:				**0.038**
– Western Cape	5.5 (149)	5.1 (73)	5.9 (76)	
– Eastern Cape	13.6 (371)	15.3 (219)	11.8 (152)	
– Northern Cape	7.3 (200)	7.5 (107)	7.2 (93)	
– Free State	9.9 (270)	10.1 (145)	9.7 (125)	
– Kwa-Zulu Natal	18 (489)	18.2 (261)	17.7 (228)	
– North West	8.9 (242)	7.7 (111)	10.2 (131)	
– Gauteng	8.5 (231)	7.4 (106)	9.7 (125)	
– Mpumalanga	12.4 (338)	12.4 (178)	12.4 (160)	
– Limpopo	15.9 (433)	16.4 (235)	15.4 (198)	
Education level				**<0.0001**
– Primary or less	10.4 (283)	13.2 (190)	7.2 (93)	
– Secondary complete	84.1 (2289)	86 (1234)	81.9 (1055)	
– Tertiary	5.6 (151)	0.8 (11)	10.9 (140)	
Employed	13.2 (358)	3.5 (50)	23.9 (308)	**<0.0001**
Wealth index				0.205

– Quintile 1 (Poorest)	23.3 (634)	24.9 (357)	21.5 (277)	
– Quintile 2 (Poorer)	23.5 (640)	22.2 (318)	25 (322)	
– Quintile 3 (Middle)	23.1 (630)	22.9 (328)	23.5 (302)	
– Quintile 4 (Richer)	19.8 (540)	19.7 (282)	20 (258)	
– Quintile 5 (Richest)	10.3 (279)	10.5 (150)	10 (129)	

*Variables tested using Chi-squared.

Table S3. Prevalence of single disease conditions by sex and method of measurement in South Africa for 2016 (weighted data)

Disease condition		Total %(95% CI)	Male %(95% CI)	Female %(95% CI)
**Self-reported**	Diabetes	0.22 (0.09–0.52)	0.25 (0.07–0.89)	0.18 (0.07–0.45)
	Bronchitis/COPD	0.27 (0.14–0.53)	0.14 (0.03–0.55)	0.43 (0.2–0.91)
	Heart disease	0.51 (0.29–0.88)	0.28 (0.12–0.67)	0.77 (0.38–1.54)
	High cholesterol	0.47 (0.2–1.09)	0.12 (0.03–0.48)	0.89 (0.35–2.27)
	Stroke	0.1 (0.04–0.28)	0.07 (0.01–0.51)	0.14 (0.04–0.41)
	TB (in past 12 months)	0.47 (0.26–0.84)	0.45 (0.2–1.01)	0.5 (0.22–1.13)
**Measured**	**HIV**	**8.37 (6.8–10.27)**	**3.73 (2.35–5.87)**	**13.71 (10.88–17.15)**
	Hypertension	18.56 (16.33–21.03)	20.24 (17.44–23.36)	16.62 (13.54–20.24)
	Anaemia	23.27 (20.98–25.73)	13.26 (10.6–16.46)	34.38 (30.68–38.27)
	Diabetes	1.61 (1.04–2.49)	1.98 (1.1–3.55)	1.19 (0.68–2.09)
**Self-reported & measured**	Diabetes	1.18 (0.78–1.76)	1.47 (0.85–2.51)	0.83 (0.49–1.41)

Table S4. Prevalence of single disease conditions by age group and method of measurement in South Africa for 2016 (weighted data)

Disease condition		Total %(95% CI)	15–19 year old %(95% CI)	20–24 year olds %(95% CI)
**Self-reported**	Diabetes	0.22 (0.09–0.52)	0.19 (0.04–0.92)	0.24 (0.1–0.6)
	Bronchitis/COPD	0.27 (0.14–0.53)	0.34 (0.14–0.82)	0.2 (0.07–0.54)
	Heart disease	0.51 (0.29–0.88)	0.37 (0.12–1.14)	0.65 (0.37–1.14)
	High cholesterol	0.47 (0.2–1.09)	0.2 (0.06–0.65)	0.76 (0.27–2.11)
	Stroke	0.1 (0.04–0.28)	0.04 (0.01–0.19)	0.17 (0.05–0.55)
	TB (in past 12 months)	0.47 (0.26–0.84)	0.35 (0.14–0.91)	0.6 (0.28–1.25)
**Measured**	HIV	8.37 (6.8–10.27)	5.65 (4–7.93)	11.36 (8.75–14.63)
	Hypertension	18.56 (16.33–21.03)	12.74 (10.46–15.41)	24.83 (21.21–28.84)
	Anaemia	23.27 (20.98–25.73)	25.01 (21.9–28.4)	21.38 (17.73–25.55)
	Diabetes	1.61 (1.04–2.49)	1.3 (0.73–2.29)	1.96 (1.08–3.51)
**Self-reported & measured**	Diabetes	1.18 (0.78–1.76)	1.47 (0.85–2.51)	0.83 (0.49–1.41)

Table S5. Number of diseases in individuals by age group in South Africa for 2016 (weighted data)

Number of diseases	Total % (95% CI)	15–19 year olds % (95% CI)	20–24 year olds % (95% CI)
No disease	69.64 (67.11–72.05)	71.62 (68.55–74.51)	67.53 (63.66–71.18)
1 disease	25.13 (23.04–27.35)	25.36 (22.51–28.44)	24.89 (22.04–27.97)
2 diseases	4.14 (3.37–5.07)	2.77 (1.99–3.84)	5.6 (4.26–7.33)
3 diseases	1.09 (0.65–1.81)	0.25 (0.1–0.68)	1.98 (1.12–3.47)
**Multimorbidity (≥ 2 diseases)**	5.23 (4.25–6.41)	3.02 (2.21–4.11)	7.58 (5.9–9.67)

Table S6. Number of MEASURED diseases in individuals by age group in South Africa for 2016 (weighted data)

Number of diseases	Total % (95% CI)	15–19 year olds % (95% CI)	20–24 year olds % (95% CI)
No disease	70.71 (68.20–73.10)	72.72 (69.67–75.57)	68.57 (64.71–72.2)
1 disease	24.55 (22.46–26.76)	24.62 (21.79–27.68)	24.48 (21.65–27.55)
**Multimorbidity (≥ 2 diseases)**	**4.47 (3.79–5.92)**	**2.66 (1.92–3.68)**	**6.95 (5.31–9.04)**

Note: MM here is defined as only measured disease (i.e. self-reported is excluded).

Table S7. Number of diseases in individuals by sex in South Africa for 2016 (weighted data)

Number of diseases	Total % (95% CI)	Male % (95% CI)	Female % (95% CI)
No disease	69.64 (67.11–72.05)	74.97 (71.79–77.9)	63.34 (60–66.56)
1 disease	25.13 (23.04–27.35)	22.6 (19.9–25.55)	28.12 (25.26–31.17)
2 diseases	4.14 (3.37–5.07)	2.28 (1.52–3.42)	6.33 (4.96–8.04)
3 diseases	1.09 (0.65–1.81)	0.15 (0.04–0.52)	2.2 (1.2–3.80)
**Multimorbidity (≥ 2 diseases)**	5.23 (4.25–6.41)	2.43 (1.65–3.58)	8.53 (6.65–10.9)

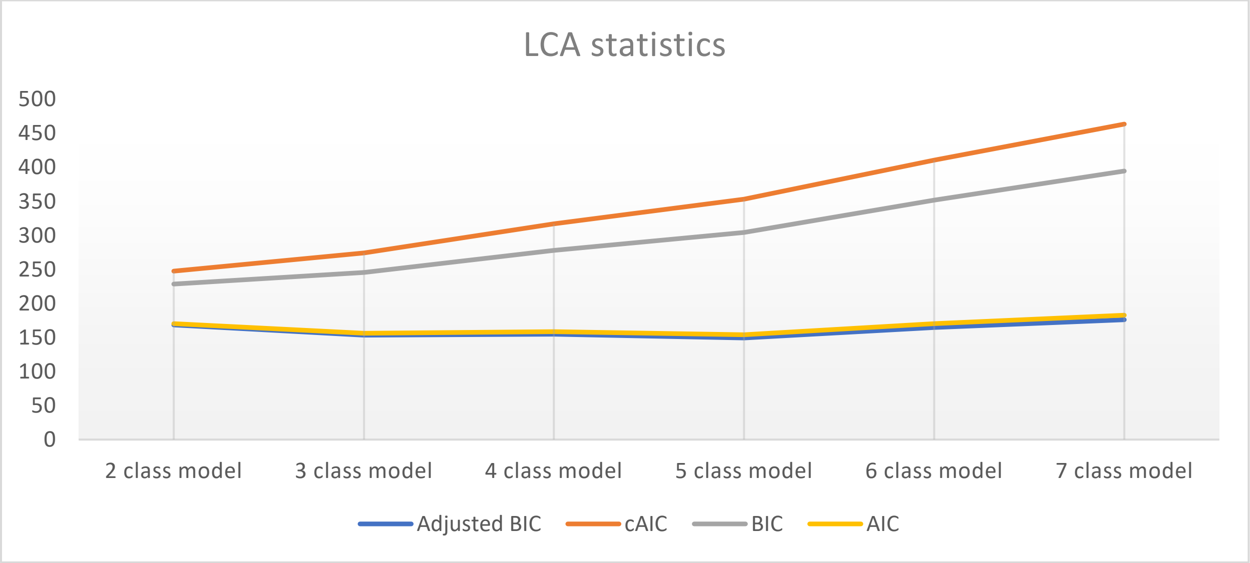

Figure S1. LCA statistics

Table S8. Item response probabilities, by disease for each class

Class	Class name	Item response probability								
		**HIV**	**Diabetes**	**Hyper-tension**	**Anae-mia**	**Bronch-itis**	**Heart disease**	**Choles-terol**	**Stroke**	**TB**
Class 1	Diabetes, Anaemia	34%	**97%**	35%	**71%**	0%	0%	0%	0%	5%
Class 2	HIV, Anaemia	**100%**	0%	37%	**100%**	0%	6%	0%	0%	1%
Class 3	HIV, Hypertension	**100%**	0%	**97%**	16%	0%	0%	0%	0%	8%
Class 4	Hypertension, Anaemia	2%	0%	**100%**	**100%**	0%	0%	15%	0%	0%
Class 5	Hypertension, Heart disease	12%	8%	**68%**	**36%**	22%	**41%**	14%	18%	18%
